# Effects of Mouthguards on Skin Damage In Vitro Study

**DOI:** 10.1055/s-0042-1756474

**Published:** 2022-10-28

**Authors:** Maho Saito, Kazunori Nakajima, Arata Tsutsui, Takahiro Sakaue, Anna Kanemitsu, Tomotaka Takeda, Kenichi Fukuda

**Affiliations:** 1 Division of Sports Dentistry, Department of Oral Health and Clinical Science Dentistry, Tokyo Dental College, Tokyo, Japan; 2 Division of Special Needs Dentistry and Orofacial Pain, Department of Oral Health and Clinical Science Dentistry, Tokyo Dental College, Tokyo, Japan

**Keywords:** mouthguard, prevention, skin, thickness, trauma

## Abstract

**Objective** Mouthguards can prevent and reduce orofacial sports traumas, which occur to the players themselves. However, the effect of mouthguards on skin damage has not been clarified. The present study's purpose was to examine whether the mouthguard can reduce or prevent skin damage caused by teeth (including the difference in mouthguard thickness).

**Materials and Methods** Pigskins, artificial teeth, and Ethylene-vinyl acetate (EVA) mouthguard blanks with 1.5- and 3.0-mm thickness were employed. Each of the two type mouthguards was produced in 10 replicates. Mouthguard incisal thickness and collision touch angle were measured on a PC using imaging software. A pendulum-type machine was used to apply impact. Strain gauges attached to the tooth and impacted plate were used to measure mouthguards' effect on impact stress. Also, a microscope was used to observe the after impacted skin condition, and the extent of damage was assessed as a score.

**Results** The pigskin was ruptured in without mouthguard (NOMG) with presenting the highest damage score, whereas the complete rupture was not seen in the 1.5 mm MG, but the damage of the skin (defeat) was observed. No tissue change was found with the 3 mmMG. In both the flat plate and impact tooth strain, no significant difference was observed between NOMG and 1.5 mmMG. However, 3 mmMG had a significantly smaller value than the other two conditions. These results are likely to be strongly influenced by the mouthguard incisal thicknesses and collision touch angles differences.

**Conclusion** The present study results clarified that two different thickness mouthguards reduced the skin damage, and the thicker mouthguard showed more effectiveness. Therefore, mouthguards may prevent the wearer's stomatognathic system's trauma and avoid damage to the skin of other athletes they are playing with. This effect seems to be an essential basis for explaining the necessity of using mouthguards for others besides full-contact sports.

## Introduction

The effects of mouthguards for trauma prevention and reduction have been well recognized. 1 2 3 4 5 6 7 8 9 As a valuable epidemiological study, the ADA Council reported the mouthguards' value in reducing sports-related injuries to the teeth and soft tissues. 7 Further, Fernandes et al 1 revealed in their review that the prevalence of dentoalveolar trauma among mouthguard users was 7.5%, while the prevalence among non-users was 59.48%, in top quality problem-free studies. Under these circumstances, athletes and sports officials, especially in contact sports, have deepened their understanding of the importance of mouthguards, and mouthguards usage appears to have spread in the high-risk full-contact sports. However, players' teeth not covered by mouthguards might have caused injuries to other players with whom they are playing. Kaur et al mentioned those injuries have occurred even in many sports. 10 Athletes' teeth have caused traumas to allies and opponents as a weapon. 11 Prevention of these injuries is very critical as well.

However, the traumas to allies and opponents caused by other players' teeth have been seldom considered epidemiologically and experimentally. 11 If a tooth is damaged during a collision with another player, skin damage may occur in the opponent. In trauma to a head and face, an aesthetic and mental impairment may be considered. Skin damage caused by another person' s teeth resulted in infection as well. One example 12 is a soccer player who received a 3-cm laceration of the right eyebrow by an opponent's teeth. On the third day after the injury, he had a fever in the 38°C range with a headache and felt sick, and a large amount of pus was discharged from the injured part. Further, a human “bite” wound caused by a blow from a fist to another person's teeth has their specific injury pattern known as reverse bite injury, clenched fist injury, or fight bite. 13 Twenty-four cases of osteomyelitis of the hand after human bite were reviewed, 14 and almost of all the clenched-fist injuries showed a tooth mark in the bone or cartilage at the injection site with showing initial infection of the soft tissues or joint with a secondary infection of the bone. Bacteriologic studies commonly showed mixed conditions with skin and oral flora. Clenched-fist bite wounds result from direct contact of the fist on incisor teeth and are associated with polymicrobial infections. 15

The risk of infection depends on the nature and site of the wound and on individual patient characteristics, and the species by which they were bitten. 13 Rothe et al 13 reported that a bite could transmit unusual pathogens from the saliva into the wound. After a bite, the risk of infection is 10 to 20%, including 30 to 50% of cat bites, 5 to 25% of dog bites, 20 to 25% of human bites, and approximately 30 to 60% of the infections are of mixed aerobic–anaerobic origin. In the United States, 16 approximately 10% of all human bites will become infected in a child with a bite wound.

Skin damage caused by teeth might be related to blood–borne infections such as hepatitis and HIV as well. Clem and Borchers 17 mentioned that HIV/AIDS is considered a worldwide pandemic. The sports medicine physician must be aware of the risk of HIV/AIDS in the athlete. Peterson et al 18 reported that infections include human immunodeficiency virus, hepatitis C virus, and common in contact sport athletes. Anish 19 mentioned that viral hepatitis causes substantial morbidity and mortality in the general population. Most athletes who contract viral hepatitis become exposed away from the playing field as well. Further, the athlete with an immunocompromised host should be considered carefully. 20

Hence, the skin traumas caused by allies and opponents' teeth and subsequent complication in sports should be reduced and avoided as much as possible. For that purpose, it should be considered effective to use an appropriate mouthguard. However, to the best of our knowledge, there are no experimental reports on mouthguards' effect on skin damage. The aim of this study was to examine whether the mouthguard can reduce or prevent skin damage caused by a tooth (including the difference in mouthguard thickness) using a system including a pendulum-type impact tester.

## Materials and Methods

Pigskins were employed in the current study. Skin samples were prepared from pig foot, which is commercially available in a frozen state. After thawing in the refrigerator for 24 hours, the pigskin was cut into squares of 20 mm in length and width and 2.0 ± 0.3 mm in thickness. The pigskins used in this study include epidermis and dermis. 21 22 Before the experiment, pigskins were left in a laboratory at 25°C for 2 hours to ensure it was thawed before use. This time was determined in a preliminary experiment. The thawing was confirmed by a Shore hardness tester (Durometer 200, Shimadzu, Kyoto, Japan) with a value of approximately 36.

**Fig. 1:**
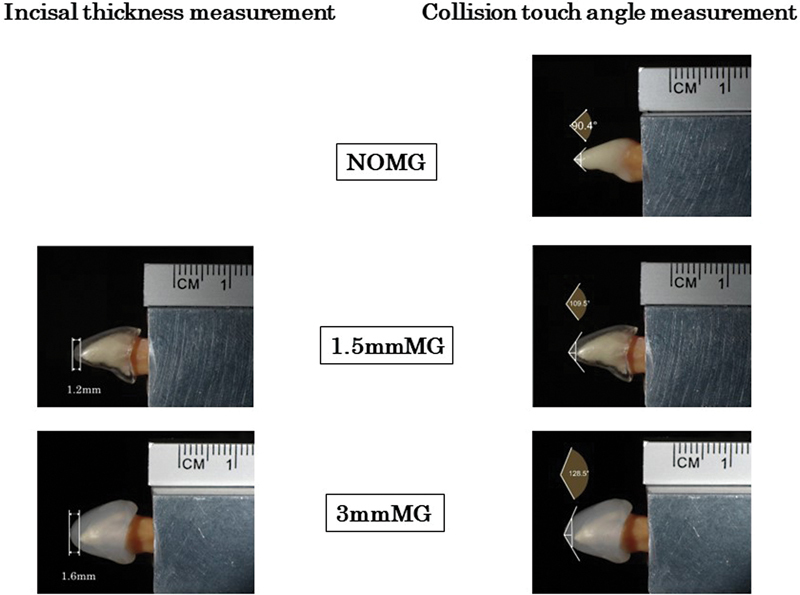
*Mouthguard incisal thickness and collision touch angle measurements.* Mouthguard incisal thickness and collision touch angle for each specimen were measured on a PC using imaging software.

Prepared pigskins were changed for each impact. An artificial tooth (Upper right central incisor, B2–306: Nissin, Kyoto, Japan) was used as an impact object. An impression made with alginate was taken to make a plaster model for the fabrication of the different mouthguards. Clear Ethylene-vinyl acetate (EVA) mouthguard blanks with a Shore hardness 82 (1.5 and 3.0 mm, YAMAHACHI DENTAL MFG, CO, Aichi, Japan) were used in mouthguards production. An air pressure type machine (Drufomat Scan Dreve-Dentamid GMBH, Unna, Germany) was used for thermoforming the mouthguard materials. A heating time of 111 and 135 seconds, manufacture's recommended conditions, and an approximate 0.6 MPa air pressure were employed. Hereafter, two types of tested mouthguards were coded 1.5 mmMG (actual incisal edge thickness was approximately 1.1 mm, Table 1 .) and 3 mmMG (1.8-mm thickness); also, without mouthguard (NOMG). Mouthguard incisal thickness and collision touch angle for each specimen (artificial tooth, 1.5, and 3.0 mmMG) were measured on a PC using imaging software (Dartfish 9 TeamPro, 9.0: Dartfish Japan Co, Ltd., Tokyo, Japan) ( Fig. 1 ). The images were taken under the same conditions at equal magnification from the mesial plane. In that case, a metallic measuring ruler was incorporated into the images. The mouthguard incisal edge thickness was determined by the distance between the end of the mouthguard and the tip of the tooth, as compared with the measure. To determine the collision touch angle, a perpendicular line to the tooth axis was first drawn 1 mm from the artificial tooth's tip or the mouthguards. Next, the intersection points between the line and the tooth or the mouthguards outline were found above and below. The collision touch angle was the angle between the two lines connecting those points and the tip. And the collision touch angle was measured three times for each sample. Each of the two types was produced in 10 replicates. The impact tests described below were performed for each pair of intact pigskin and a new mouthguard.

**Table 1:** Mouthguard incisal thickness (mm)

A.				
	1.5 mmMG		3 mmMG	
Mean	1.1		1.8	
S.D.	0.2		0.1	
**B. Student's unpaired** ***t*** **-test**				
**Level 1**	**Level 2**	***t*** **-Value**	**d** ***f***	***p***
1.5 mmMG	3 mmMG	15.045	29	0.000

**Fig. 2:**
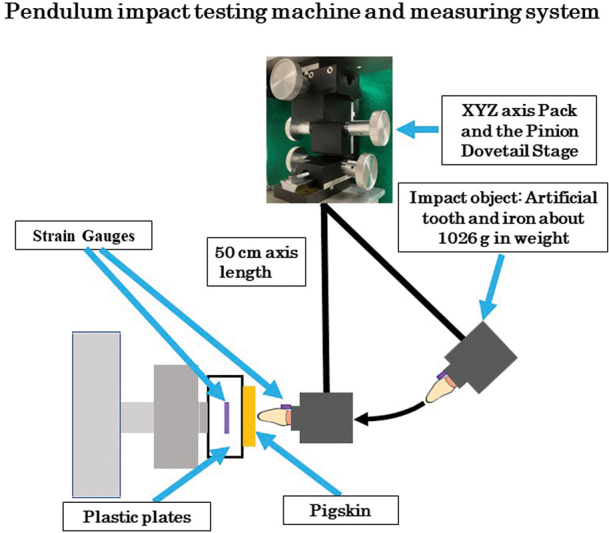
*Pendulum impact testing machine and measuring system.* The customized device used in the current study consisted of an impact iron object with an artificial tooth and plastic plates attached strain gauges to measure the shock absorbing ability of the mouthguard. The impact point was adjusted using the XYZ axis Pack and the Pinion Dovetail Stage.

A pendulum impact testing machine 23 24 with an approximately 50 cm axis length and an impact object of approximately 1,026 g in weight iron ingot with the artificial tooth planted in an aluminum frame with a resin (UNIFAST III No. 8, GC corp. Tokyo, Japan). In the process, the tooth axis was fixed at a right angle to the colliding surface. A strain gauge (KFG-1N-120-C1–11N15C2: Kyowa, Tokyo, Japan) was put on the labial cervical surface of the artificial tooth with the same direction of impact force. And two bonded flat-topped, round acrylic plastic plates of 50 mm diameter and 15 mm height (a strain gauge: KFG-1N-120-C1–11L1M2R had fixed on the backside of the outer plastic plate center with the perpendicular direction to the impact force, and another plate was put on the surface firmly) were used to measure transmitted forces as strains ( Fig. 2 ). The impact point was adjusted using the XYZ axis Pack and the Pinion Dovetail Stage (TAR-70135, Sigma Koki, Tokyo, Japan) attached to the axial point of the pendulum arm so that the impact object was able to accurately impact upon the center of the plastic plates vertically ( Fig. 2 ). The prepared pigskins were put on the plastic surface (four corners were fixed with double-sided tape) except for the control trial. The impact distance used was 15 cm from the tooth surface. This distance was determined to be the maximum distance at which the impact tooth would not fracture in preliminary experiments. The acceleration at that time was approximately 45 G. This value was set as about twice the value obtained from an Instrumented accelerometer in mouthguards against head impacts in amateur rugby. 25 Mechanical forces recorded by the strain gages were amplified, converted into an electric voltage output, and stored as data with a memory recorder analyzer (EDX-1500A, Kyowa). The data was then analyzed with the Data Analysis Software (DAS-100A, Kyowa). The mean value and standard deviation were calculated for each variable.

A Skybasic 50 X to 1000 X Wifi Handheld Zoom Magnification Endoscope (Skybasic, China) was used to observe the after-impact skins condition. After staining the skins with India ink for 5 seconds and washing with running water for 10 seconds, the skin was observed from both the surface and the backside to evaluate the degree of damage. Three dentists made this evaluation, and where there was a difference in evaluation, the majority value was taken. Each sample of the skin was blinded so that no one could see to which group it belonged. Each evaluation score was set; 0 points for no damage, 1 point for surface only, and 2 points for both sides (penetration).

Statistical analysis was performed using the Student's unpaired *t* -test for the incisal thickness of mouthguards, Kruskal–Wallis test for the after-impact skin condition score, and one-way ANOVA and the Tukey multiple comparison test ( *p* -value as <0.001) for the collision touch angle, the strain on the plastic plate, and the impact tooth in the SPSS Statistics 25.0 software package (IBM, Tokyo, Japan). Further, Steel-Dwass multiple comparison tests ( *p* -value as <0.001) for the after-impact skin condition was performed in the BellCurve for Excel (Social Survey Research Information Co., Ltd., Tokyo, Japan).

## Results

The actual thickness of each mouthguard at the incisal edge was on Table 1A . Significant difference ( *p* -value as <0.001) was observed between 1.5and 3 mmMG ( Table 1B ).

Each collision touch angle was on Table 2A . Statistical analysis ( Table 2B , one-way ANOVA, *p* -value as <0.001) revealed significant differences between the two types of mouthguards and NOMG. Significant differences ( *p* -value as <0.001) were observed among all three conditions ( Table 2C ).

**Table 2:** Collision touch angle (degree)

A.					
	NOMG		1.5 mmMG	3 mmMG	
Mean	88.2		110.5	135.3	
SD	2.7		2.7	5.7	
**B. One-way ANOVA**					
	**Sum of squares**	**D** ***f***	**Mean square**	***F***	***p***
Between groups	33337.055	2	16668.527	1064.051	0.000
Within groups	1362.868	87	15.665		
Total	34699.923	89			
**C. Tukey multiple comparison test**					
**Level 1**		**Level 2**		***p***	
NOMG		1.5 mmMG		0.000	
NOMG		3 mmMG		0.000	
1.5 mmMG		3 mmMG		0.000	

**Fig. 3:**
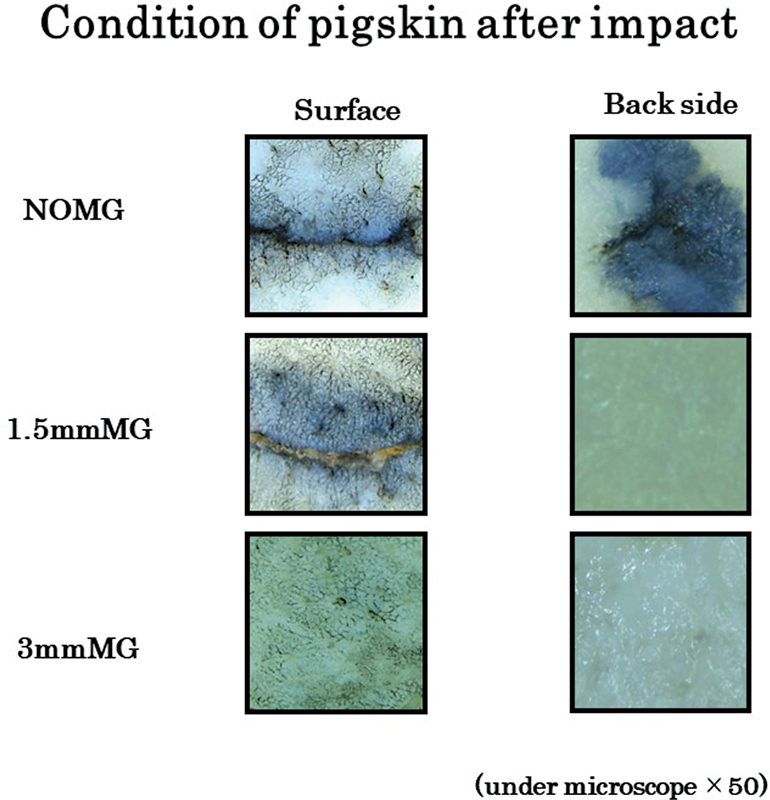
*Condition of pigskin after impact with microscope.* Both the surface and the backside tissues were ruptured in NOMG, whereas when the 1.5 mmMG was attached, complete backside rupture of the tissue was not seen, but surface damage of the skin was observed. No tissue change was observed with the 3 mmMG.

In the after-impact pigskin, it was possible to confirm the condition under the naked eye and the microscope. Both the surface and the backside tissues were ruptured (penetration) was confirmed for most samples in NOMG. In contrast, when 1.5 mmMG was attached, the tissue's complete backside rupture was not seen, but the skin's surface damage was observed. No tissue change was observed with the 3 mmMG ( Fig. 3 ). Each score of after-impact pigskin conditions was on Table 3A . Kruskal–Wallis test, ( *p* -value as <0.001) revealed significant differences between the two types of mouthguards and NOMG ( Table 3B ). Significant differences ( *p* -value as <0.001) were observed among all three conditions ( Table 3C ).

**Table 3:** Pigskin evaluation score

A.			
	NOMG	1.5 mmMG	3 mmMG
Mean	1.8	1	0
SD	0.4	0	0
**B. Kruskal Wallis test**			
Kruskal-Wallis H(K)		D *f*	*p*
26.583		2	0.000
**C. Steel-Dwass test**			
**Level 1**	**Level 2**	**Statistic**	***p***
NOMG	1.5 mmMG	3.559	0.001
NOMG	3 mmMG	4.1944	0.00008
1.5 mmMG	3 mmMG	4.3289	0.00004

Score

0: No damage was found on the skin

1: Damage was found on the surface but not on the backside

2: Damage was found on both the surface and the backside

**Fig. 4:**
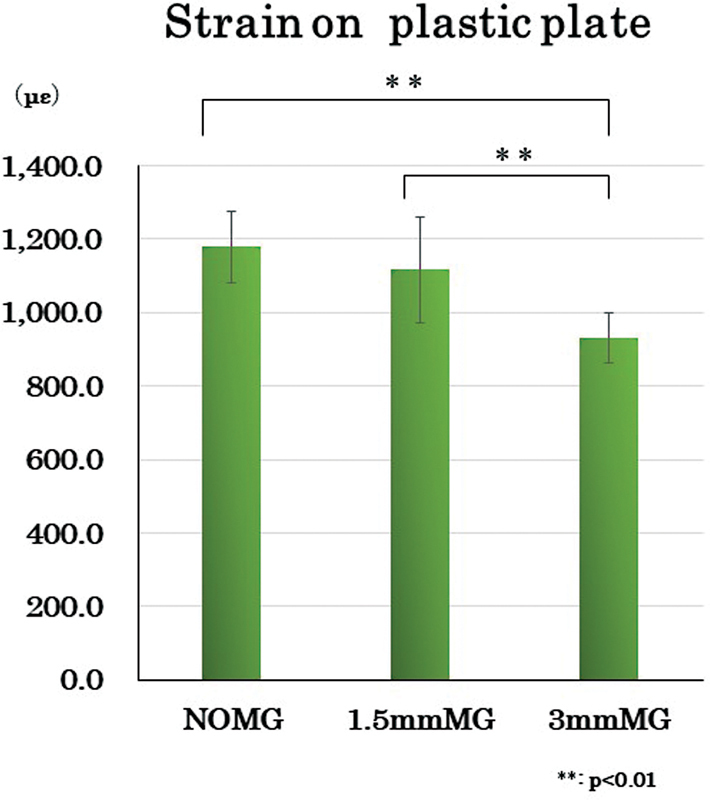
*Strain on plastic plate* . No significant difference was observed between NOMG and the 1.5 mmMG, but 3 mmMG showed a significantly smaller value than the other two conditions. Also, a significant difference was observed between 1.5 and 3 mmMG.

Statistical analysis ( Table 4A , one-way ANOVA, *p* -value as <0.001) revealed significant differences between the two types of mouthguards and NOMG. Fig. 4 presents the strain on the plastic plate with the Tukey multiple comparison test results ( Table 4B ). As a result of the strain (με) of the plastic flat plate, no significant difference ( *p* -value as <0.001) was observed between NOMG (1,178.8 ± 98.5) and the 1.5 mmMG (1,116.5 ± 143.6), but 3 mmMG (930.6 ± 69.0) showed a significantly smaller value than NOMG ( *p* -value as <0.001). Also, significant difference ( *p* -value as <0.001) was observed between 1.5and 3 mmMG.

**Table 4:** Strain on plastic plate

A. One-way ANOVA					
	Sum of squares	d *f*	Mean square	*F*	*p*
Between groups	333,337.139	2	166,668.569	14.238	0.000
Within groups	316,062.280	27	11,706.010		
Total	649,399.419	29			
**B. Tukey multiple comparison test**					
**Level 1**	**Level 2**	**Statistic**		***p***	
NOMG	1.5 mmMG	1.2867		0.4146	
NOMG	3 mmMG	5.1283		0.00006	
1.5 mmMG	3 mmMG	3.8416		0.0019	

**Fig. 5:**
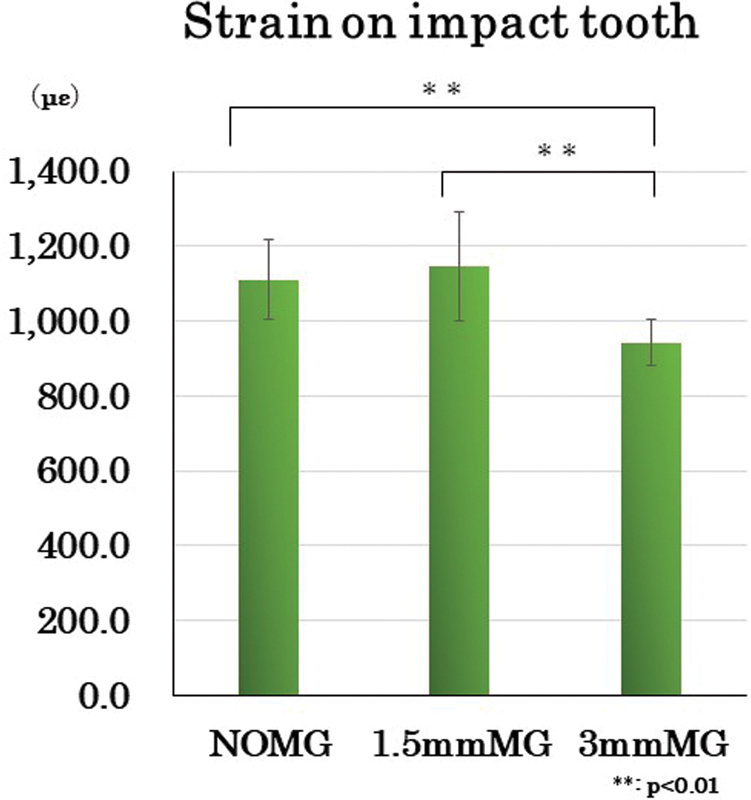
*Strain on impact tooth.* No significant difference was observed between NOMG and 1.5 mmMG, but 3 mmMG showed a significantly smaller value than the other two conditions. Also, a significant difference was observed between 1.5 and 3 mmMG.

Statistical analysis ( Table 5A , one-way ANOVA, *p* -value as <0.001) revealed significant differences among the two types of mouthguards and NOMG. Fig. 5 presents the strain on the impact tooth with the Tukey multiple comparison test results ( Table 5B ). As a result of the strain (με) of the impact tooth, no significant difference ( *p* -value as <0.001) was observed between NOMG (1,110.7 ± 104.7) and 1.5 mmMG (1,146.0 ± 145.6), but 3 mmMG (942.1 ± 62.4) showed a significantly smaller value than NOMG ( *p* -value as <0.001). Also, significant difference ( *p* -value as <0.001) was observed between 1.5 and 3 mmMG.

**Table 5:** Strain on impact tooth

A. One-way ANOVA					
	Sum of squares	d *f*	Mean square	*F*	*p*
Between groups	237,535.317	2	118,767.658	9.881	0.001
Within groups	324,522.525	27	12,,019.353		
Total	562,,057.842	29			
**B. Tukey multiple comparison test**					
**Level 1**	**Level 2**	**Statistic**		***p***	
NOMG	1.5 mmMG	0.719		0.7545	
NOMG	3 mmMG	3.4398		0.0052	
1.5 mmMG	3 mmMG	4.1587		0.0008	

## Discussion

In the present study, in both the flat plate and impact tooth strain, no significant difference was observed between NOMG and 1.5 mmMG. However, 3 mmMG had a significantly smaller value than the other two conditions. Furthermore, thinking over the facts that NOMG showed the tissue rupture by presenting the highest damage score, 1.5 mmMG showed only surface damages, and 3 mmMG showed no tissue damage. These results are likely to be strongly influenced by the mouthguard incisal thicknesses differences (1.1 mm of 1.5 mmMG and 1.8 mm of 3.0 mmMG) and collision touch angles differences (approximately 88 of artificial tooth, 111 of 1.5 mmMG, and 135 degrees of 3.0 mmMG). In NOMG, much of the impact energy was consumed as destructive energy of the skin damage. In 1.5 mmMG, due to two effects: a certain amount of the impact energy absorption by the mouthguard material itself, and conversion of the impact energy into the destructive energy at the time of skin damage reduced the impact energy. Therefore, it is considered that both strains were equivalent in NOMG and 1.5 mmMG. In 3 mmMG, the material thickness itself has a high impact absorption capacity. 3 mmMG showed the minimum strain, although the skin damage did not occur. These results suggest that mouthguards are useful in preventing damage to the skin, and the higher the thickness, less is the damage to the skin.

The reasons for the employment of pigskin in this study; due to its similarity in skin anatomy and physiology, the pig appears to be a well-suited animal model for preclinical studies of skin analog transplantations 21 and models to test tissue-engineered skin in injuries. 22 Pigskin similarities to human skin in terms of its anatomical structure are, epidermis thickness in pigs ranges from 30 to 140 μm in pigs, and from 50 to 120 μm in humans, both show similar dermal collagen and similar dermal–epidermal thickness ratio. 21 Further, the pigskin is safe (edible), non-infectious, and stable in supply. About the artificial tooth employment, a preliminary experiment was conducted using bovine and extracted human teeth. However, both teeth easily broke after several impacts, even with the mouthguard attached. Therefore, the artificial tooth made of plastic was used. Besides, when the impact distance and impact force were more extensive than those of this experiment, the artificial tooth was damaged in NOMG.

The increased mouthguard thickness effect on safety (due to shock absorption ability) agrees with many previous studies. 26 27 The present study on the skin also supports these findings. Moreover, it is considered that the effect of mouthguard on the pigskin might be due to the increase in collision touch angle at the time of impact in addition to the thickness increase. Wong et al 28 mentioned that in sharp force trauma, there is a localized breaching of the skin layer coupled with the wedging action of the impacting object. In the present study, NOMG as the sharp instrument caused an open injury (incised wound). On the other hand, 1.5 mmMG as a blunt instrument seems to have caused another open injury (contused wound).

The maxillary front teeth often receive most of the direct impacts. More than 90% of sports-related dental injuries are contained in this area. 29 30 Lakshmi et al 31 reported that the prevalence of tooth fractures was 8.7% ( *n* = 628), and most tooth fractures had occurred due to tripping or slipping (62.4%). On investigating the specific reason for tooth fracture, collision against the object was the most frequent cause, followed by a fall from stairs or a bicycle. Clashes against an item might include a human being. These traumas of anterior teeth fracture may cause skin injury and subsequent complications if the viewpoint is changed.

It is said that a condition in an oral cavity influences the frequency of oral trauma. Patients who need orthodontics treatment are more affected by trauma. Bauss et al 32 mentioned that in 1,367 patients for orthodontic treatment, the most frequently affected teeth were the maxillary central incisors. The frequency of dental trauma was significantly higher in patients with increased overjet and adequate lip coverage or increased overjet and inadequate lip coverage. A significant percentage of orthodontic treatment candidates, especially those with increased overjet and insufficient lip coverage, suffer trauma to their permanent incisors before the onset of orthodontic treatment. Patients who need orthodontics are particularly vulnerable and dangerous. Mouthguards should be provided to these patients. For that reason, most orthodontic departments' consultants routinely advise the use of a custom-made mouthguard to patients wearing fixed equipment while playing contact sports. 33

Recently, the use of mouthguards has increased. However, this is since the usage rate of mouthguards seems to be high in compulsory sporting events. Recognition and wearing rate of mouthguards are different in non-mandatory sporting events. Galic et al 4 mentioned that there was a significant difference in the use of mouthguards between taekwondo (73.7%) and karate (70.7%) players (mandatory sports) compared with handball (14.5%) and water polo players (5.1%) (non-mandatory sports). Zamora-Olave et al 6 also mentioned that 10.1% of water polo players had tried a mouthguard; however, only 1.2% used it habitually even though 57.9% of them reported at least one orofacial injury in a season. Even if it is not a so-called full-contact sport such as soccer, volleyball, basketball, or handball, which require many jumps during competitions, there is a chance of collision with the head or other body part of an opponent at the timing of landing, etc. It is during this time that trauma might be caused by a collision with an opponent's tooth. So, the use of mouthguards should be strengthened, including in such competitions to reduce skin damage and subsequent infections.

This paper has certain limitations. The present study was just a simulated experimental study that used pigskins, artificial teeth (no periodontal ligament), the rigid plastic plates, the two types of mouthguards, and a pendulum-type testing machine. The present experiment is a physical two-dimensional analysis, therefore it may be necessary to investigate the possibility of using three-dimensional methods such as the finite element method. Further, a large-scale survey will be necessary for the near future. We sports dentists must also promote the understanding and spread of proper mouthguards other than in contact sports to reduce and prevent many serious skin injuries and infections. Suitable mouthguards, especially with the appropriate thickness, could be beneficial in the trauma in allies and opponents caused by players' incisal teeth (as a kind of incise weapon).

## Conclusion

The present study results clarified that two different thickness mouthguards reduced the skin damage, and the thicker mouthguard showed more effectiveness. Therefore, mouthguards may prevent the wearer's stomatognathic system's trauma and avoid damage to the skin of other athletes they are playing with. This effect seems to be an essential basis for explaining the necessity of using mouthguards for others besides full-contact sports. Also, in this sense, the mouthguard must be provided with an appropriate thickness.
